# Thyroid Hormone Induces Oral Cancer Growth via the PD-L1-Dependent Signaling Pathway

**DOI:** 10.3390/cells11193050

**Published:** 2022-09-29

**Authors:** Kuan-Wei Su, Hung-Yun Lin, Hsien-Chung Chiu, Shin-Yu Shen, Chun A. ChangOu, Dana R. Crawford, Yu-Chen S. H. Yang, Ya-Jung Shih, Zi-Lin Li, Haw-Ming Huang, Jaqueline Whang-Peng, Yih Ho, Kuan Wang

**Affiliations:** 1 Department of Dentistry, Hsinchu MacKay Memorial Hospital, Hsinchu City 30071, Taiwan; 2 Graduate Institute of Cancer Molecular Biology and Drug Discovery, College of Medical Science and Technology, Taipei Medical University, Taipei 11031, Taiwan; 3 Cancer Center, Wan Fang Hospital, Taipei Medical University, Taipei 11031, Taiwan; 4 TMU Research Center of Cancer Translational Medicine, Taipei Medical University, Taipei 11031, Taiwan; 5 Traditional Herbal Medicine Research Center of Taipei Medical University Hospital, Taipei Medical University, Taipei 11031, Taiwan; 6 Pharmaceutical Research Institute, Albany College of Pharmacy and Health Sciences, Rensselaer, NY 12144, USA; 7 Department of Periodontology, School of Dentistry, National Defense Medical Center and Tri-Service General Hospital, Taipei 11490, Taiwan; 8 Core Facility, Taipei Medical University, Taipei 11031, Taiwan; 9 Department of Immunology and Microbial Disease, Albany Medical College, Albany, NY 12208, USA; 10 Joint Biobank, Office of Human Research, Taipei Medical University, Taipei 11031, Taiwan; 11 Graduate Institute of Nanomedicine and Medical Engineering, College of Medical Engineering, Taipei Medical University, Taipei 11031, Taiwan; 12 School of Dentistry, College of Oral Medicine, Taipei Medical University, Taipei 11031, Taiwan; 13 School of Pharmacy, Taipei Medical University, Taipei 11031, Taiwan

**Keywords:** thyroxine, oral cancer, PD-L1, β-catenin

## Abstract

Oral cancer is a fatal disease, and its incidence in Taiwan is increasing. Thyroid hormone as L-thyroxine (T_4_) stimulates cancer cell proliferation via a receptor on integrin αvβ3 of plasma membranes. It also induces the expression of programmed death-ligand 1 (PD-L1) and cell proliferation in cancer cells. Thyroid hormone also activates β-catenin-dependent cell proliferation in cancer cells. However, the relationship between PD-L1 and cancer proliferation is not fully understood. In the current study, we investigated the role of inducible thyroid hormone-induced PD-L1-regulated gene expression and proliferation in oral cancer cells. Thyroxine bound to integrin αvβ3 to induce PD-L1 expressions via activation of ERK1/2 and signal transducer and activator of transcription 3 (STAT3). Inactivated STAT3 inhibited PD-L1 expression and nuclear PD-L1 accumulation. Inhibition of PD-L1 expression reduced β-catenin accumulation. Furthermore, nuclear PD-L1 formed a complex with nuclear proteins such as p300. Suppression PD-L1 expression by shRNA blocked not only expression of PD-L1 and β-catenin but also signal transduction, proliferative gene expressions, and cancer cell growth. In summary, thyroxine via integrin αvβ3 activated ERK1/2 and STAT3 to stimulate the PD-L1-dependent and β-catenin-related growth in oral cancer cells.

## 1. Introduction

Oral cancer has the fourth highest malignant prevalence in males and accounts for the seventh highest level in the general population of Taiwan [1]. Oral cancer is a fatal disease, and its incidence has been increasing in Taiwan [2]. About 95% of oral cancers in Taiwan are oral squamous cell carcinomas (OSCCs). Around 50% of new OSCC cases found in medical centers present with stage III or IV cancer lesions which leads to low 5-year survival [1]. The thyroid hormone was shown to affect the progression of different types of cancers including oral cancers [3,4]. However, mechanisms of the thyroid hormone in oral cancer are not fully understood. Changes in thyroid hormone receptor expression were associated with oral cancer progression. Expression of thyroid receptor β1 (TRβ1) in biopsy tissues of oral cancer patients was reduced compared to that in normal mucosa [5,6]. Expression of the thyroid hormone cell surface receptor integrin, αvβ3, in tumor vascular epithelial cells was associated with decreased survival and increased metastatic potential of oral cancer [7]. Furthermore, L-thyroxine (T_4_) was shown to increase cell numbers per crypt in thyroidectomized rats [5]. Therefore, blocking thyroid hormone signaling may be beneficial for treating oral cancer.

The αvβ3 integrin is a structural protein of plasma membranes primarily expressed by rapidly proliferating cells, namely, dividing blood vessel cells [2] and cancer cells [8,9]. The protein is essential for interactions of these cells with extracellular matrix proteins and growth factors [10]. The thyroid hormone binds to αvβ3 [11,12,13] to stimulate its translocation into the cytosol where it activates extracellular signal-regulated kinase 1/2 (ERK1/2) phosphorylation. The internalized integrin αv monomer complexes with phosphorylated (p)-ERK1/2 to translocate into nuclei and bind to thyroxine-responsive gene promoters as a co-activator to activate transcriptional events, such as tumor cell proliferation [1] and angiogenesis [13]. Studies also indicated that T_4_ potentiates epidermal growth factor (EGF) action, but inhibits the effect in transforming growth factor (TGF)-α-treated cells [14,15]. Thus, there may be crosstalk between thyroid hormone and growth factor receptor signaling pathways in certain cancer cells.

The programmed cell death protein 1 (PD-1)/programmed cell death ligand 1 (PD-L1) checkpoint plays an essential role in cancer growth. PD-1/PD-L1 are important regulators of the interaction between activated T cells and tumor cells [13]. The PD-1/PD-L1 checkpoint protects tumor cells against immune surveillance. The PD-L1 protein is expressed by tumor cells and binds to PD-1, thereby reversing the interaction between activated T cells and tumor cells. Additionally, the ligand may also serve to induce apoptosis of T cells. Increased PD-L1 expression was positively correlated with cancer progression. Overexpression of PD-L1 may be correlated with cancer patient mortality. However, a report suggested that PD-L1 expression promotes the prognosis of locally advanced oral squamous cell carcinoma [14]. Activation of phosphatidylinositol 3 kinase (PI3K) and signaling pathways of signal transduction and activator of transcription 3 (STAT3) are essential for PD-L1 gene expression in cancer cells [16,17,18,19]. It was shown that overexpression of PD-1/PD-L1 is significantly associated with pSTAT3 in human and murine head and neck squamous cell carcinoma (HNSCC). Activation of STAT3 was significantly correlated with PD-1/PD-L1 overexpression in human and mouse HNSCCs [20]. Recently, our studies revealed that thyroxine induces PD-L1 expression and its protein accumulation in different cancer types [21,22]. ERK1/2 activation is crucial for thyroxine-induced PD-L1 expression [22]. On the other hand, inhibition of PI3K activity was able to suppress PD-L1 expression and reduced tumor growth in vitro and in a xenograft murine model [19]. PD-L1 was detected in nuclei, with an unknown function [3]. These results suggest that upregulation of PD-L1 may play a vital role in cancer proliferation.

Expression of PD-L1 is β-catenin-dependent in glioblastoma [23]. Although β-catenin was originally determined to be a cell-cell adhesion component, it can interact with the cytoplasmic domain of E-cadherin and link E-cadherin to α-catenin [24]. Wnt signaling also modulates cytosolic β-catenin phosphorylation/degradation [7,25]. Development of several cancer types was linked to increased β-catenin transcriptional activity that results from Wnt/Wingless-dependent or -independent signaling [26,27]. Furthermore, a mutant Wnt pathway often leads to upregulated levels of β-catenin, which is a major indicator of neoplastic processes in oral cancers. On the other hand, studies showed that Wnt/β-catenin signaling induces high mobility group AT-hook 2 (HMGA2) expression [28]. We showed that the thyroid hormone modulates β-catenin expression and induces β-catenin nuclear accumulation [28]. Thyroxine promotes β-catenin-directed transactivation of CCND1 and c-Myc in colorectal cancer cells [12,25]. In addition, overexpression of HMGA2 is able to elicit the epithelial-to-mesenchymal transition (EMT) and regulate several genes which are closely related to the Wnt/β-catenin pathway by directly binding to their promoter, thereby activating the Wnt/β-catenin pathway [29].

Against this background, in the current study, we investigated the mechanisms involved in thyroxine-induced PD-L1 expression via integrin αvβ3-activated signal transduction pathways in oral cancer cells. Activation of ERK1/2 and STAT3 was essential for PD-L1 expression. Nuclear PD-L1 promoted β-catenin expression and collaborated with nuclear β-catenin to stimulate gene expressions. Sequentially, proliferation was assumed in human oral cancer cells. We demonstrated that PD-L1 expression was induced by thyroxine through integrin αvβ3, activated ERK1/2, and STAT3-dependent pathways. Consequently, it resulted in binding of nuclear PD-L1 and p300 complexes to induce proliferative gene expressions. Thyroxine binding to integrin αvβ3 blockage, inactivation of ERK1/2 or STAT3, and PD-L1 depletion inhibited phosphorylation of ERK1/2 and p38 in tumor cells. Consequently, suppression of oral cancer growth occurred.

## 2. Materials and Methods

### 2.1. Cell Cultures

Human oral epidermoid carcinoma OEC-M1 cells were a gift from Dr. Hsien-Chung Chiu (Department of Periodontology, School of Dentistry, National Defense Medical Center, Taipei, Taiwan). Human squamous carcinoma of the tongue SCC-25 cells (ATCC^®^ CRL-1628™) were obtained from the American Type Culture Collection (ATCC^®^; Manassas, VA, USA). SCC-25 cells were authenticated (by an isoenzyme analysis, Mycoplasma, cytogenetics, tumorigenesis, and receptor expression testing) by the Bioresource Collection and Research Center (BCRC; Hsinchu, Taiwan). Cells were maintained in RPMI-1640 (OEC-M1 cells) or Dulbecco’s modified Eagle medium (DMEM)/F12 (SCC-25 cells) supplemented with 10% fetal bovine serum (FBS) in an incubator with 5% CO_2_ at 37 ºC and then used for experiments until passage 40. Thyroid hormone affected gene expression and protein accumulation in oral cancer cells. For thyroxine (T_4_) concentrations study, oral cancer cell line, OEC-M1 or SCC-25 cells, were set in 6 wells tray of about 40% confluence and incubated overnight. Before T_4_ treatment, cells were incubated in serum-free medium for 2 days. After starvation, cells were refed medium with 5% hormone-stripped FBS. Cells were treated with different concentrations of T_4_ (10^−8^ M to 10^−6^ M) (Sigma–Aldrich, Saint Louis, MO, USA) for 24 h. Cells were harvested for either Western blotting analyses or qPCR assay.

### 2.2. Confocal Microscopy

OEC-M1 and SCC-25 cells were exponentially grown on sterilized cover glasses (Paul Marienfeld, Lauda–Königshofen, Germany) and treated with 10^−7^ M T_4_ in the presence or absence of a STAT3 inhibitor, S31-201 (Sigma, SML0330) for another 24 h. Samples were immediately fixed with 4% paraformaldehyde in PBS for 10 min. Cells were permeabilized in 0.1% Triton X-100 in PBS for 20 min. After 1 h of 1% BSA blocking, cells on the slides were incubated with an anti-PD-L1 (GeneTex, San Antonio, TX, USA, GTX104763) and P300 (GeneTex, GTX30618) antibody overnight at 4 °C. Then, cells were incubated with secondary antibody conjugated with Alexa Fluor 647 (abcam, Cambridge, UK, ab150079), Alexa Fluor 488 (GeneTex, GTXGTX213110-04) or Alexa Fluor 594 (GeneTex, GTX213111-05) and DAPI (ThermoFisher Scientific, Waltham, MASS, USA, S36938) stain for nuclei. The fluorescent signals of PD-L1 or P300 were recorded and analyzed with a TCS SP5 Confocal Spectral Microscope Imaging System (Leica Microsystems, Wetzlar, Germany). The figures shown are representative of four fields for each experimental condition.

### 2.3. Transfection of Short Hairpin (sh)RNA

Oral cancer OEC-M1 cells were seeded (10^5^ cells/well) in six-well tissue culture plates, grown overnight, and maintained in the absence of antibiotics for 24 h before transfection. Just prior to transfection, the culture medium was removed, and cells were washed once with phosphate-buffered saline (PBS) and then transfected with *PD-L1* shRNA (CD274, clone ID: TRCN0000056916) or a scrambled plasmid (TRC1.Scramble, clone ID: ASN0000000004) (0.5 µg/well, RNAi Core Facility, Academia Sinica, Taipei, Taiwan) by Lipofectamine 3000 Kit (ThermoFisher Scientific, L3000001) in Opti-MEM I medium according to the manufacturer’s instructions. After transfection, cultures were incubated at 37 °C for 6 h and then placed in fresh culture medium for an additional 48 h. Transfected cells were then treated with agents for qPCR (24 h), Western blot experiments (24 h) or cell proliferation assays (72 h).

### 2.4. Real-Time Quantitative Polymerase Chain Reaction (qPCR)

After treatment, cells were washed with PBS and total RNA was extracted with a NucleoSpin RNA, RNA purification Mini Kit (MACHEREY-NAGEL, MN-740955.50). Total RNA (1 μg) was reverse-transcribed using a RevertAid H Minus First Strand cDNA Synthesis Kit (ThermoFisher Scientific, K1632) into complementary (c)DNA. cDNAs were used as templates for the real-time PCR and analysis. Real-time PCRs were conducted using a QuantiNovaTM SYBR^®^ Green PCR Kit (Qiagen, Hilden, Germany, QIA-208056) on a CFX Connect™ Real-Time PCR Detection System (Bio-Rad Laboratories, Hercules, CA, USA). The reaction protocol was initially denatured at 95 °C for 5 min, followed by 40 cycles of denaturing at 95 °C for 5 s and combined annealing/extension at 60 °C for 10 s as detailed in the manufacturer’s instructions. The relative gene expression (normalized to the *18S* reference gene) was calculated according to the ΔΔCT method. The fidelity of the PCR was determined by a melting temperature analysis. Primer sequences of genes of interest are listed in Table 1. Calculations of the relative mRNA expressions (normalized to the *18S* reference gene) were performed according to the ΔΔCT method. Fidelity of the PCR was determined with a melting temperature analysis.

### 2.5. Western Blot Analysis

For the Western blot analyses, cells were lysed, and extracted protein samples were separated by 10% sodium dodecyl sulfate polyacrylamide gel electrophoresis (SDS-PAGE). A 15-μg quantity of protein was loaded into each well with 5× sample buffer, and samples were separated by electrophoresis at 100 V for 2 h. Separated proteins were transferred from the polyacrylamide gel to Millipore Immobilon-PSQ Transfer polyvinylidene difluoride (PVDF) membranes (Millipore, Billerica, MA, USA) using Mini Trans-Blot^®^ Cell (Bio-Rad Laboratories, Hercules, CA, USA). Membranes then were incubated in NaCl/Tris blocking buffer containing 2% bovine serum albumin (BSA). Membranes were incubated with primary antibodies for PD-L1 (GeneTex, GTX104763), β-catenin (BD, #610153), pβ-catenin (Cell Signaling, Danvers, MASS, USA, #9561), pSTAT3 (Cell Signaling, #9136), pERK1/2 (Cell Signaling, #4377), ERK1/2 (Cell Signaling, #9102), or GAPDH (proteintech, 60004-1-Ig) overnight at 4 °C. After 0.1% TBST washed, proteins were detected with horseradish peroxidase (HRP)-conjugated secondary antibodies (Jackson ImmunoResearch, West Grove, PENN, USA, #115-035-003 and #111-035-003) and the Immobilon™ Western HRP Substrate Luminol Reagent (Millipore, WBKLS0500). Western blots were visualized and recorded with an Amersham Imager 600 (GE Healthcare Life Sciences, Pittsburgh, PA, USA). The densitometric analysis of Western blots was conducted using ImageJ 1.47 software (National Institutes of Health, Bethesda, MD, USA) according to the software instructions.

### 2.6. Cell Viability Assay

After transfection of shRNA, cells were treated with T_4_ for 72 h. Cell viability was determined by using the Alamar Blue^®^ Assay Kit (ThermoFisher Scientific, DAL1100). At the time of detection, medium was removed, and cells were incubated with Alamar Blue^®^ reagent for 2 h at 37 °C according to the manufacturer’s instructions. Plates were then analyzed using a VersaMax Microplate reader (Molecular Devices, San Jose, CA, USA) at a wavelength of 570 nm, with 600 nm as a reference.

### 2.7. Statistical Analysis

In this study, all of the collected data of immunoblot and nucleotide densities were analyzed by IBM^®^SPSS^®^ Statistics software vers. 19.0 (SPSS, Chicago, IL, USA). Two-tailed Student’s *t*-test was conducted and considered significant at *p* < 0.05 (* or #), < 0.01 (** or ##) and <0.001 (*** or ###).

## 3. Results

### 3.1. Thyroxine Induces PD-L1 and β-Catenin Expressions in Human Oral Cancer Cells

**Figure 1:**
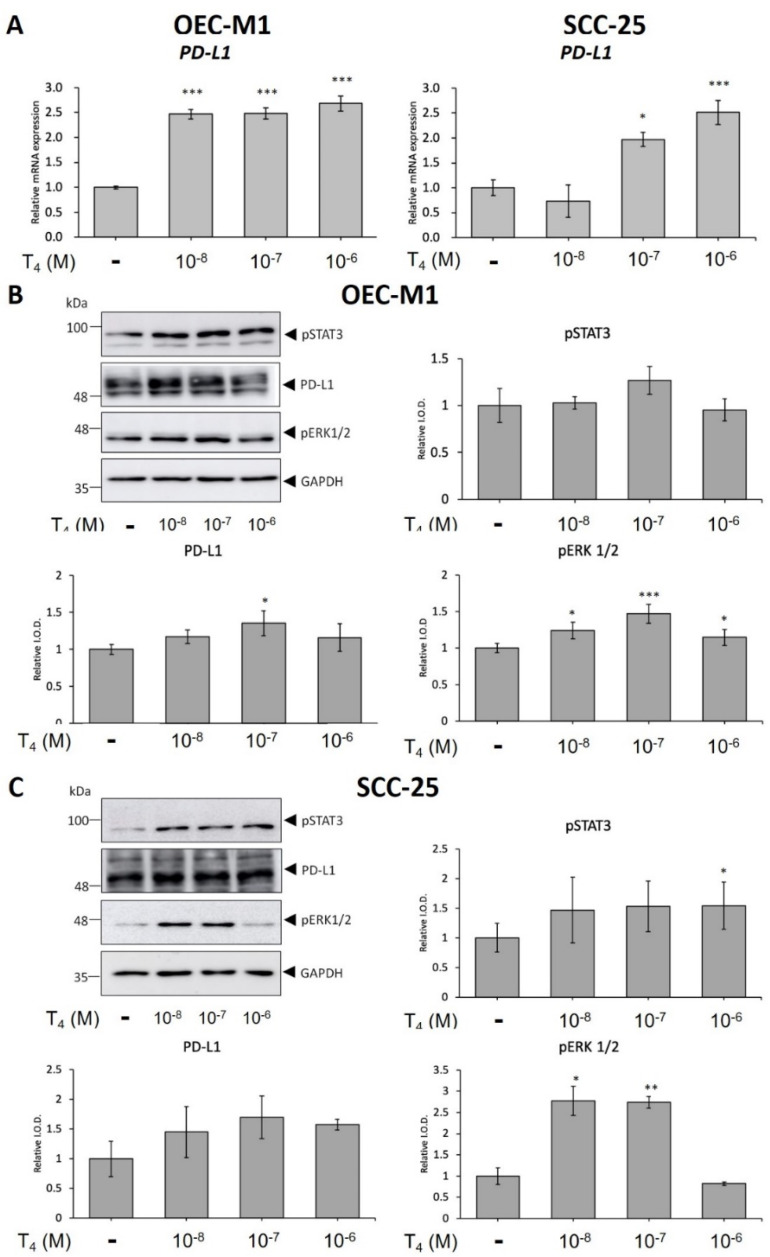
**Thyroid hormone induces abundances of PD-L1 in human oral cancer cells.** OEC-M1 cells and SCC-25 cells were treated with different concentrations of thyroid hormone (T_4_, 10^-8^ to 10^-6^ M) for 24 h. (**A**) Thyroid hormone-induced *PD-L1* expression in both oral cancer cell. (**B**) OEC-M1 cells and (**C**) SCC-25 cells were treated with different concentrations of T_4_ for 24 h. Cells were harvested. Total protein was extracted and Western blot analyses of pSTAT3, pERK1/2, PD-L1 were performed in Data were presented as the mean ± SD. Results are from three independent studies (*n* = 3). * *p* < 0.05, ** *p* < 0.01, *** *p* < 0.001, compared to the control. I.O.D., intensity of the optical density.

Recently, we showed that inducible PD-L1 accumulation stimulates cancer growth in colorectal cancer cells [19]. In order to investigate the role of thyroid hormone-induced PD-L1 expression in the proliferation of oral cancer cells, human oral cancer OEC-M1 cells and SCC-25 cells were treated with different concentrations of T_4_ (10^−8^ to 10^−6^ M) for 24 h. Cells were harvested and total RNA was extracted for qPCR of PD-L1 expression. Results presented in Figure 1 A indicated that thyroxine induced PD-L1 expression in both cancer cell line. Parallel studies were conducted for effect of thyroid hormone-induced signal transduction and PD-L1 accumulation in OEC-M1 cells. After 24 h treatment of thyroxine, cells were harvested and proteins were extracted. Western blot analyses were conducted for p-ERK1/2, p-STAT3, and PD-L1. Thyroxine increased phosphorylation of ERK1/2 and PI3K in concentration-dependent manners (Figure 1B). In addition, thyroxine at 10^−7^ M induced maximal PI3K activation (Figure 1B). Thyroid hormone induced significant accumulation of PD-L1 in concentration-dependent manners.

Parallel studies were conducted to investigate the role of the thyroid hormone in signal transduction in another oral cancer cell line, SCC-25 cells. SCC-25 cells were treated with different concentrations of T_4_ (10^−8^ to 10^−6^ M) for 24 h. Cells were harvested, and cytosolic and nuclear proteins were separated. Thyroxine increased activation of ERK1/2 and PI3K. In addition, thyroxine promoted the accumulation of PD-L1 compared to the untreated control (Figure 1C).

### 3.2. Activation of ERK1/2 and STAT3 Involve in Thyroid Hormone-Induced Accumulation of PD-L1 and β-Catenin and Proliferation in Oral Cancer Cells

**Figure 2:**
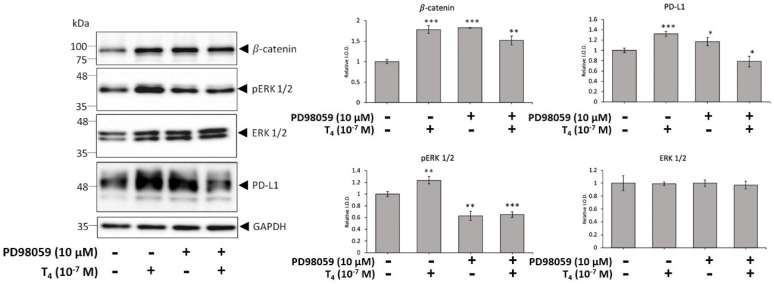
**ERK1/2 activation plays a role in T_4_-induced accumulation of PD-L1 and β-catenin in human oral cancer OEC-M1 cells.** Oral cancer OEC-M1 cells were seeded in six-well trays and were treated with 10^-7^ M T_4_ in the presence or absence of PD98059 (10 μM) for 24 h. Western blot analyses of β-catenin, PD-L1, pERK1/2 and ERK1/2 were conducted. Data are presented as the mean ± SD. Results are from three independent studies (*n* = 4). * *p* < 0.05, ** *p* < 0.01, *** *p* < 0.001, compared to the control. I.O.D., intensity of the optical density.

Thyroxine-induced PD-L1 accumulation is integrin αvβ3-signal pathway-dependent in breast cancer and colorectal cancer cells. We investigated if ERK1/2 plays role in thyroxine-induced PD-L1 expression in oral cancer cells. Oral cancer cells seeded in six-well trays were treated with 10^−7^ M T_4_ in the presence and absence of PD98059, a specific MEK antagonist, (10 µM) for 24 h. Proteins were extracted and Western blot analyses were conducted to examine pERK1/2, pPI3K, β-catenin, pβ-catenin, and PD-L1 (Figure 2). Thyroxine induced accumulation of PD-L1 and β-catenin that was activated ERK1/2-dependent. However, β-catenin phosphorylation may not be activated ERK1/2-dependent. PD98059 only inhibited ERK1/2 phosphorylation. Constitutively phosphorylated β-catenin and PD-L1 accumulation were not affected by ERK1/2 activation.

### 3.3. Thyroid Hormone Induced PD-L1 Expression

**Figure 3:**
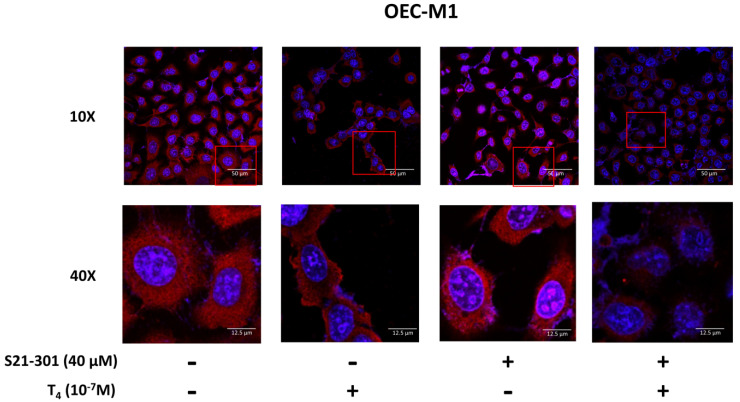
**Thyroxine (T_4_)-induced PD-L1 accumulation is activated-STAT3-dependent in human oral cancer OEC-M1 cells.** Oral cancer OEC-M1 cells were seeded on a cover glass. Cells were starved for 48 h, fed hormone-stripped serum-containing medium, and then treated with T_4_ in the presence and absence of 40 μM S31-201 for another 24 h. Cells were fixed for confocal microscopic analysis of PD-L1 accumulation (red color). Nuclei were counterstained with DAPI (blue color). The field of view of the red square is magnified for easy viewing.

**Figure 4:**
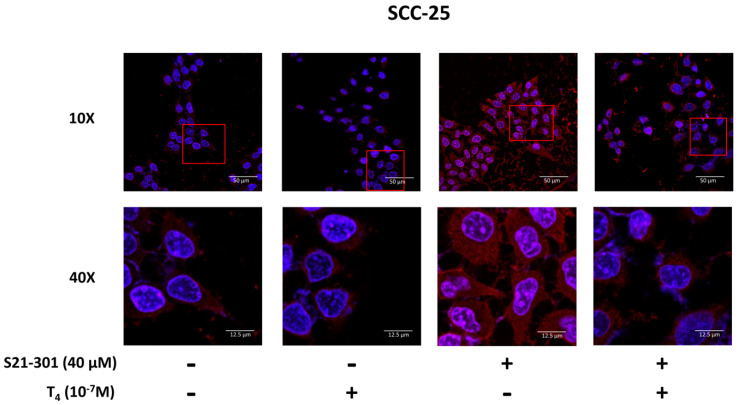
**Thyroxine (T_4_)-induced PD-L1 accumulation is activated- STAT3-dependent in human oral cancer SCC-25 cells.** Oral cancer SCC-25 cells were seeded on a cover glass. Cells were starved for 48 h, fed hormone-stripped serum-containing medium, and then treated with T_4_ in the presence and absence of 40 μM S31-201 for another 24 h. Cells were fixed for confocal microscopic analysis of PD-L1 accumulation (red color). Nuclei were counterstained with DAPI (blue color). The field of view of the red square is magnified for easy viewing.

In addition to activated ERK1/2, downstream STAT3 might play a vital role in PD-L1 accumulation. We investigated the mechanisms involved in thyroid hormone-induced PD-L1 nuclear accumulation in oral cancer cells. SCC-25 cells were pretreated with S31-201 (40 μM), a STAT3 inhibitor, for 30 min and then treated with T_4_, for another 24 h. Confocal microscopic studies were conducted. Cells were starved for 48 h, then refed hormone-stripped serum-containing medium. Cells were treated with 10^−7^ M of T_4_ for another 24 h. Cells were stained with an anti-PD-L1 antibody. In confocal microscopy, more thyroxine-treated cells exhibited nuclear PD-L1 accumulation compared to untreated nuclear PD-L1 accumulation in OEC-M1 cells (Figure 3) and SCC-25 cells (Figure 4). A specific inhibitor of STAT3 (S31-201) reduced nuclear PD-L1 accumulation in T_4_-treated cells compared to control cells. These results suggested that T_4_-induced PD-L1 accumulation was via activation of the STAT3 signaling pathway.

### 3.4. Inhibition of PD-L1 Signaling Disrupts the Thyroid Hormone-Induced Nuclear Protein Complex in Human Oral Cells

**Figure 5:**
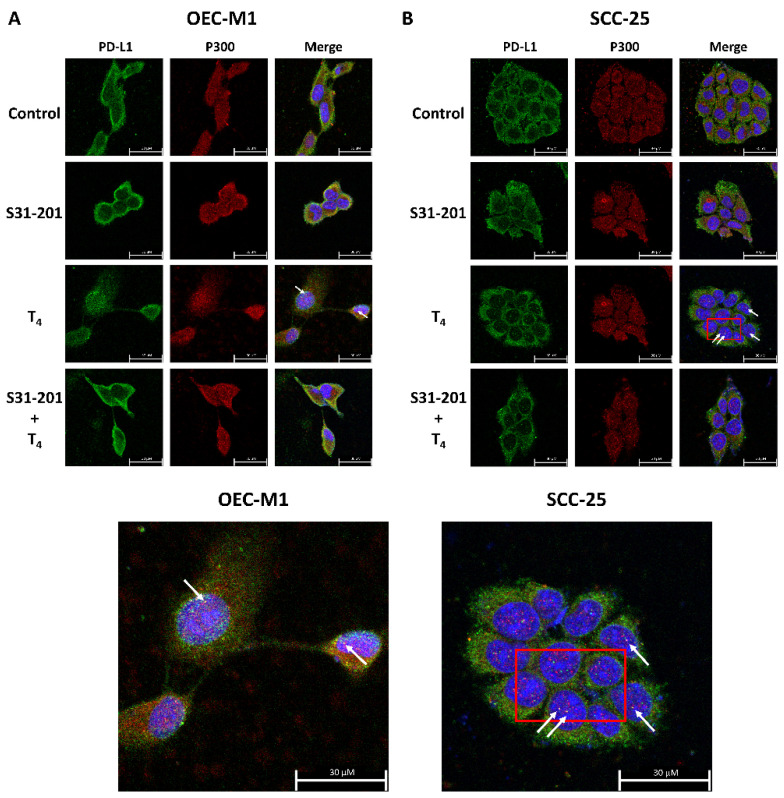
**Thyroxine (T_4_)-induced PD-L1 complexes with nuclear proteins.** Oral cancer (**A**) OEC-M1 cells and (**B**) SCC-25 cells were seeded on a cover glass. Cells were starved for 48 h, fed hormone-stripped serum-containing medium, and then treated with T_4_ in the presence and absence of 40 μM S31-201 for another 24 h. Cells were fixed for confocal microscopic analysis of PD-L1 accumulation (green color) or P300 (red color). Nuclei were counterstained with DAPI (blue color). Red square is PD-L1 and P300 co-accumulation (white arrow.). Confocal microscopy was conducted as described.

Oral cancer cells seeded in six-well trays were refed with 5% hormone-stripped FBS-containing medium and treated with thyroxine (10^−7^ M) in the presence and absence of 40 µM S31-201 for 24 h. Confocal microscopy was conducted to demonstrate the colocalization of p300 and PD-L1 in T_4_-treated oral cancer cells. After starvation, cells were refed with 5% hormone-stripped FBS-containing medium and treated with thyroxine (10^−7^ M) in the presence and absence of S31-201, a STAT3 inhibitor, for 24 h. Confocal microscopy was conducted (Figure 5). Thyroxine induced nuclear colocalization of PD-L1 and p300 which was inhibited by the STAT3 inhibitor, S31-201. These results indicated that T_4_-induced nuclear accumulated PD-L1 is able to associate with transcriptional cofactor p300 and may have functional activities in the nuclei.

### 3.5. Inhibition of PD-L1 Expression Disrupts Thyroid Hormone-Induced Gene Expressions in Human Oral Cancer Cells

**Figure 6:**
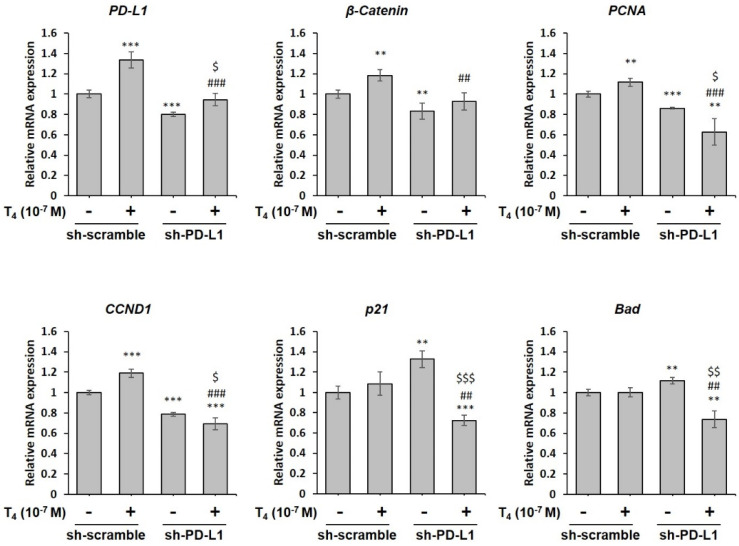
**Thyroid hormone (T_4_)-induced PD-L1 controls β-catenin expression in human oral cancer cells.** OEC-M1 cells were seeded in six-well trays and transfected with *PD-L1* shRNA or a scrambled plasmid (0.5 μg/well) were treated with 10^−7^ M T_4_ for 24 h. Cells were harvested, and RNA was extracted. A qPCR was conducted for proliferation-related genes. Data are presented as the mean ± SD. Results are from three independent studies (*n* = 4) ** *p* < 0.01, *** *p* < 0.001, compared to the control; ## *p* < 0.01, ### *p* < 0.001, compared to T_4_, and $ *p* < 0.05, $$ *p* < 0.01, $$$ *p* < 0.001, compared to *PD-L1* shRNA group.

We further investigated if thyroxine-induced PD-L1 plays a crucial role in proliferative gene expressions and cell growth in oral cancer cells. Oral cancer OEC-M1 cells were seeded in six-well trays, transfected with either shRNA of *PD-L1* (0.5 μg/well), and treated with T_4_ for 24 h. Prior to T_4_ treatment, cells were refed with hormone-stripped FBS-containing medium. Cells were harvested and total RNA was extracted. qPCRs were conducted for *PD-L1*, *β-catenin*, *PCNA*, *CCND1* and two pro-apoptotic genes, *p21* and *Bad*. Thyroxin-stimulated *PD-L1* expression significantly shRNA *PD-L1* reduced *PD-L1* expression not only in untreated control and in T_4_-treated OEC-M1 cells (Figure 6). T_4_ induced *β-catenin* expression and shRNA *PD-L1* down-regulated *β-catenin* expression compared scramble RNA with T_4_ treatment. Both *PCNA* and *CCND1* were induced by T_4_ and shRNA *PD-L1* down-regulated both gene expressions significantly. On the other hand, two pro-apoptotic genes, *p21* and *Bad* were not activated by T_4_. shRNA of *PD-L1* enhanced their expression and T_4_ treatment suppressed the expression significantly. These results suggested that *PD-L1* may be upstream of *β-catenin* and control its expression.

### 3.6. Thyroid Hormone (T_4_)-Induced PD-L1-Dependent Protein Expression and Cell Proliferation in Oral Cancer Cells

**Figure 7:**
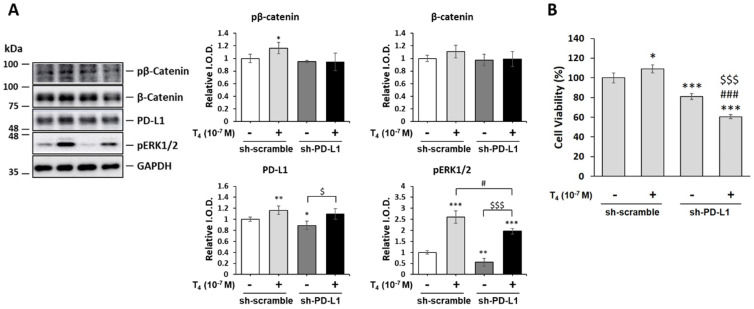
**Thyroid hormone (T_4_)-induced PD-L1-dependent protein expression and cell proliferation in oral cancer cells.** (**A**). Oral cancer OEC-M1 cells were seeded in six-well trays and were transfected with either *PD-L1* shRNA or a scrambled plasmid (0.5 μg/well). Prior to treatment, cells were refed with hormone-stripped FBS-containing medium and treated with T_4_ (10^−7^ M) for 24 h. Cells were harvested, and Western blot analyses of total β-catenin, pβ-catenin, PD-L1, and pERK1/2 were conducted. Data are presented as the mean ± SD. Results are from three independent studies (*n* = 4) ** *p* < 0.01, compared to the control. I.O.D., intensity of the optical density. (**B**). Oral cancer OEC-M1 cells were seeded in 24-well trays and were transfected with either *PD-L1* shRNA or a scrambled plasmid (0.1 μg/well). Prior to treatment, cells were refed with hormone-stripped FBS-containing medium and treated with T_4_ (10^−7^ M) for 72 h. Cells were harvested for cell viability assay. Data are presented as the mean ± SD. Results are from three independent studies (*n* = 6) * *p* < 0.05, ** *p* < 0.01, *** *p* < 0.001, compared to the control; # *p* < 0.05, ### *p* < 0.001, compared to T_4_, and $ *p* < 0.05, $$$ *p* < 0.001, compared to *PD-L1* shRNA group.

Furthermore, we examined if PD-L1-regulated gene expression was also shown in the thyroid-induced protein expression and cell proliferation in oral cancer cells. OEC-M1 cells were transfected with shRNA as described above. After T_4_ treatment, cells were harvested and total proteins were extracted. Western blot analyses of PD-L1, β-catenin, and pβ-catenin, were conducted. Thyroxine increased the accumulation of PD-L1 (Figure 7A). It also increased phosphorylation of β-catenin. Cells with transfected *PD-L1* shRNA not only exhibited reduced basal levels of pERK1/2 but also thyroxine-induced pERK1/2. Deletion of PD-L1 inhibited pp38 accumulation significantly (Supplementary Figure S1A). Moreover, it also increased the apoptosis markers, Caspase 3 and PARP cleaved. (Supplementary Figure S1B,C). Parallel studies were conducted for cell proliferative assay (Figure 7B). Knockdown *PD-L1* by shRNA not only suppressed cancer cell viability but also reduced T_4_-induced cancer proliferation significantly (Figure 7B).

## 4. Discussion

PD-L1 overexpression has been detected in different types of cancers including melanomas [30], pancreatic cancer [31], colorectal cancer [32], lung cancer cells [33], head and neck cancers (HNCs), and OSCC [34]. Immuno-targeted therapies against PD-L1/PD-1 were developed as immunotherapy in several types of cancer cells [35]. However, clinical outcomes showed the activities of those antibodies to be beneficial to only a minority of patients with different types of cancer [10]. The thyroid induces PD-L1 expression and its protein nuclear accumulation [21]. In addition, the thyroid hormone increases β-catenin expression in cancer cells [22]. Integrin αvβ3 is involved in thyroid hormone-induced accumulation of PD-L1 and β-catenin and the proliferation of oral cancer cells (Figure 1) [36]. The integrin αvβ3 has been shown to involve in expressions of PD-L1 [37]. These results suggested that thyroxine stimulates PD-L1 expression in oral cancer cells via an integrin αvβ3-dependent pathway. Thyroid hormone-induced PD-L1 expression and its protein accumulation is activated ERK1/2-depedent (Figure 2) [22,38] and PI3K-dependent [19]. Our results also indicated that thyroid hormone-induced PD-L1 nuclear translocation was inhibited by a STAT3 inhibitor (Figure 3 and Figure 4). Growth factors and Wnt ligands stimulate PI3K activation and loss of phosphatase and tensin homolog (PTEN) to trigger AKT activation and promote β-catenin-dependent PD-L1 expression and tumor immune evasion [23]. The AKT inhibitor, MK2206, currently in clinical trials for treating cancer [39], was shown to suppress expression levels of PD-L1 in tumor cells, enhance cluster of differentiation-positive (CD8+) T-cell infiltration, and inhibit tumor growth. β-catenin activation induced by both Wnt ligand and activated EGF receptor (EGFR) results in the binding of the β-catenin/transcription factor (TCF)/lymphoid enhancer-binding factor (LEF) complex to the promoter region of the *CD274* gene to induce PD-L1 expression [40] transcription factor (TCF) [41]. Depletion of β-catenin reduces PD-L1 expression in tumor cells [23]. However, our results indicated that suppression of *PD-L1* expression also reduced *β-catenin* expression (Figure 6) and β-catenin levels (Figure 7A). Previous research show that inhibited p38 signaling reduced the proliferation of oral cancer cells [42] and HNSCC cells both in vitro and in vivo [43]. PD-L1 knockdown by shRNA decreased the expressions of proliferation genes and increased apoptosis genes (Figure 6). In addition, it not only inhibited the accumulation of pERK1/2 (Figure 7A) and pp38 (Supplementary Figure S1A) but also increased the cleavage of Caspase 3 and PARP (Supplementary Figure S1B,C). It demonstrated that deletion of PD-L1 inhibited cell proliferation (Figure 7B) and induced apoptosis in oral cancer cells.

Recent studies conducted by Yang Gao et al. indicated that PD-L1 is translocated from plasma membranes into nuclei through interactions with components of endocytosis and nucleocytoplasmic transport pathways [44]. p300-mediated acetylation and histone deacetylase 2 (HDAC2)-dependent deacetylation of PD-L1 control the transport process [44]. Thyroxine increased nuclear accumulation of PD-L1 (Figure 3 and Figure 4). Results of confocal microscopy revealed that thyroxine-induced nuclear-accumulating PD-L1 colocalized with p300 (Figure 5). Inhibition of p300/CBP enhances the efficacy of PD-L1 blockade treatment in prostate cancer [19]. On the other hand, thyroxine induces integrin αvβ3 endocytosis. The monomer integrin, αv, forms a complex with pERK1/2 to translocate to nuclei where it further complexes with p300 as a transactivator complex [45]. Therefore, p300 may also complex with PD-L1 and other transcriptional factors to process gene expression.

Treatments targeting PD-1 and PD-L1 have been approved and have exhibited durable clinical benefits in human cancer patients [46,47]. However, the majority of cancer patients are resistant to anti-PD-1/PD-L1 immunotherapies. The fundamental resistance mechanisms remain unclear. In a large variety of cancers, EGFR activation and active Wnt signaling directly mediate the β-catenin/TCF/LEF transcriptional complex to induce PD-L1 expression [23]. Alleviation of these effects by the systemic depletion of CD8+ T cells further supports a critical role of AKT and β-catenin transactivation-regulated PD-L1 expression in tumor immunogenicity [23]. MK2206 combined with an anti-PD-1 antibody, which caused more profound abrogation of immune checkpoint blockade, significantly enhanced CD8+ T-cell infiltration, and blocked tumor growth. Upregulation of PD-L1 is controlled by widely activated Wnt signaling, EGFR, AKT, and by the frequently mutated PTEN. Active β-catenin signaling in melanoma cells promotes T-cell exclusion through decreased chemokine ligand 4 (CCL4) production by tumor cells [48]. However, the Wnt/β-catenin pathway does not affect *CCL4* gene expression in glioblastoma multiforme (GBM) cells, suggesting that CCL4 is not involved in β-catenin-promoted T-cell exclusion in the GBM microenvironment.

## 5. Conclusions

Thyroxine induced PD-L1 expression via integrin αvβ3. In addition, it also activated ERK1/2 and STAT3 phosphorylation. The nuclear accumulation of PD-L1 in oral cancer cells was activated-STAT3-dependent. Knockdown of PD-L1 inhibited the phosphorylation of ERK1/2, and p38, and reduced cancer cell proliferation. These results suggested that PD-L1 is upstream of β-catenin and modulates β-catenin’s biological activities, including β-catenin-dependent cell proliferation. This does not exclude the possibility that PD-L1 directly controls oral cancer cell proliferation as previously shown in colorectal cancer cells [19].

## Figures and Tables

**Table 1:** Primer sequences for the qPCR.

	Forward	Reverse
***PD-L1***	5′-GTTGAAGGACCAGCTCTCCC-3′	5′-ACCCCTGCATCCTGCAATTT-3′
***β-catenin***	5′-CTGGTCCTTTTTGGTCGAGGA-3‘	5′-GCAAGGCTAGGGTTTGATAAAT-3′
***CCND1***	5′-CAAGGCCTGAACCTGAGGAG-3′	5′-GATCACTCTGGAGAGGAAGCG-3′
***PCNA***	5′-TCTGAGGGCTTCGACACCTA-3′	5′-TCATTGCCGGCGCATTTTAG-3′
***p21***	5′-CTGGGGATGTCCGTCAGAAC-3′	5′-CATTAGCGCATCACAGTCGC-3′
***BAD***	5′-CTTTAAGAAGGGACTTCCTCGCC-3′	5′-AAGTTCCGATCCCACCAGGA-3′
***18S***	5’-GTAACCCGTTGAACCCCATT-3’	5’-CCATCCAATCGGTAGTAGCG-3’

## Data Availability

The original contributions presented in the study are included in the article; further inquiries can be directed to the corresponding author.

## Supplementary Materials

The following supporting information can be downloaded at: https://www.mdpi.com/article/10.3390/cells11193050/s1, Figure S1: Deletion of PD-L1 affected proliferation and apoptosis-related protein accumulation in oral cancer cells.

## References

[1] Kao S.Y., Lim E. (2015). An overview of detection and screening of oral cancer in Taiwan. Chin. J. Dent. Res..

[2] Tseng C.H. (2013). Oral cancer in Taiwan: Is diabetes a risk factor?. Clin. Oral Investig..

[3] Hou J., Zhao R., Xia W., Chang C.W., You Y., Hsu J.M., Nie L., Chen Y., Wang Y.C., Liu C. (2020). PD-L1-mediated gasdermin C expression switches apoptosis to pyroptosis in cancer cells and facilitates tumour necrosis. Nat. Cell Biol..

[4] Lin C.C., Chin Y.T., Shih Y.J., Chen Y.R., Chung Y.Y., Lin C.Y., Hsiung C.N., Whang-Peng J., Lee S.Y., Lin H.Y. (2019). Resveratrol antagonizes thyroid hormone-induced expression of checkpoint and proliferative genes in oral cancer cells. J. Dent. Sci..

[5] Brown A.R., Simmen R.C., Simmen F.A. (2013). The role of thyroid hormone signaling in the prevention of digestive system cancers. Int. J. Mol. Sci..

[6] Vonlaufen A., Wiedle G., Borisch B., Birrer S., Luder P., Imhof B.A. (2001). Integrin alpha(v)beta(3) expression in colon carcinoma correlates with survival. Mod. Pathol..

[7] MacDonald B.T., Tamai K., He X. (2009). Wnt/beta-catenin signaling: Components, mechanisms, and diseases. Dev. Cell.

[8] Chen L., Han X. (2015). Anti-PD-1/PD-L1 therapy of human cancer: Past, present, and future. J. Clin. Investig..

[9] Page D.B., Postow M.A., Callahan M.K., Allison J.P., Wolchok J.D. (2014). Immune modulation in cancer with antibodies. Annu. Rev. Med..

[10] Schmidt L.H., Kummel A., Gorlich D., Mohr M., Brockling S., Mikesch J.H., Grunewald I., Marra A., Schultheis A.M., Wardelmann E. (2015). PD-1 and PD-L1 Expression in NSCLC Indicate a Favorable Prognosis in Defined Subgroups. PLoS ONE.

[11] Lin H.Y., Tang H.Y., Keating T., Wu Y.H., Shih A., Hammond D., Sun M., Hercbergs A., Davis F.B., Davis P.J. (2008). Resveratrol is pro-apoptotic and thyroid hormone is anti-apoptotic in glioma cells: Both actions are integrin and ERK mediated. Carcinogenesis.

[12] Nana A.W., Chin Y.T., Lin C.Y., Ho Y., Bennett J.A., Shih Y.J., Chen Y.R., Changou C.A., Pedersen J.Z., Incerpi S. (2018). Tetrac downregulates beta-catenin and HMGA2 to promote the effect of resveratrol in colon cancer. Endocr. Relat. Cancer.

[13] Davis F.B., Tang H.Y., Shih A., Keating T., Lansing L., Hercbergs A., Fenstermaker R.A., Mousa A., Mousa S.A., Davis P.J. (2006). Acting via a cell surface receptor, thyroid hormone is a growth factor for glioma cells. Cancer Res..

[14] Kogashiwa Y., Yasuda M., Sakurai H., Nakahira M., Sano Y., Gonda K., Ikeda T., Inoue H., Kuba K., Oba S. (2017). PD-L1 Expression Confers Better Prognosis in Locally Advanced Oral Squamous Cell Carcinoma. Anticancer Res..

[15] Mincione G., Di Marcantonio M.C., Tarantelli C., D’Inzeo S., Nicolussi A., Nardi F., Donini C.F., Coppa A. (2011). EGF and TGF-beta1 Effects on Thyroid Function. J. Thyroid Res..

[16] Atsaves V., Tsesmetzis N., Chioureas D., Kis L., Leventaki V., Drakos E., Panaretakis T., Grander D., Medeiros L.J., Young K.H. (2017). PD-L1 is commonly expressed and transcriptionally regulated by STAT3 and MYC in ALK-negative anaplastic large-cell lymphoma. Leukemia.

[17] Jiang X., Zhou J., Giobbie-Hurder A., Wargo J., Hodi F.S. (2013). The activation of MAPK in melanoma cells resistant to BRAF inhibition promotes PD-L1 expression that is reversible by MEK and PI3K inhibition. Clin. Cancer Res..

[18] Mittendorf E.A., Philips A.V., Meric-Bernstam F., Qiao N., Wu Y., Harrington S., Su X., Wang Y., Gonzalez-Angulo A.M., Akcakanat A. (2014). PD-L1 expression in triple-negative breast cancer. Cancer Immunol. Res..

[19] Huang T.Y., Chang T.C., Chin Y.T., Pan Y.S., Chang W.J., Liu F.C., Hastuti E.D., Chiu S.J., Wang S.H., Changou C.A. (2020). NDAT Targets PI3K-Mediated PD-L1 Upregulation to Reduce Proliferation in Gefitinib-Resistant Colorectal Cancer. Cells.

[20] Bu L.L., Yu G.T., Wu L., Mao L., Deng W.W., Liu J.F., Kulkarni A.B., Zhang W.F., Zhang L., Sun Z.J. (2017). STAT3 Induces Immunosuppression by Upregulating PD-1/PD-L1 in HNSCC. J. Dent. Res..

[21] Chang T.C., Chin Y.T., Nana A.W., Wang S.H., Liao Y.M., Chen Y.R., Shih Y.J., Changou C.A., Yang Y.S., Wang K. (2018). Enhancement by Nano-Diamino-Tetrac of Antiproliferative Action of Gefitinib on Colorectal Cancer Cells: Mediation by EGFR Sialylation and PI3K Activation. Horm. Cancer.

[22] Lin H.Y., Chin Y.T., Nana A.W., Shih Y.J., Lai H.Y., Tang H.Y., Leinung M., Mousa S.A., Davis P.J. (2016). Actions of l-thyroxine and Nano-diamino-tetrac (Nanotetrac) on PD-L1 in cancer cells. Steroids.

[23] Du L., Lee J.H., Jiang H., Wang C., Wang S., Zheng Z., Shao F., Xu D., Xia Y., Li J. (2020). β-Catenin induces transcriptional expression of PD-L1 to promote glioblastoma immune evasion. J. Exp. Med..

[24] Huber A.H., Weis W.I. (2001). The structure of the beta-catenin/E-cadherin complex and the molecular basis of diverse ligand recognition by beta-catenin. Cell.

[25] Lee Y.S., Chin Y.T., Shih Y.J., Nana A.W., Chen Y.R., Wu H.C., Yang Y.S.H., Lin H.Y., Davis P.J. (2018). Thyroid Hormone Promotes beta-Catenin Activation and Cell Proliferation in Colorectal Cancer. Horm. Cancer.

[26] Schweizer L., Varmus H. (2003). Wnt/Wingless signaling through beta-catenin requires the function of both LRP/Arrow and frizzled classes of receptors. BMC Cell Biol..

[27] Gorka J., Marona P., Kwapisz O., Waligorska A., Pospiech E., Dobrucki J.W., Rys J., Jura J., Miekus K. (2021). MCPIP1 inhibits Wnt/beta-catenin signaling pathway activity and modulates epithelial-mesenchymal transition during clear cell renal cell carcinoma progression by targeting miRNAs. Oncogene.

[28] Wend P., Runke S., Wend K., Anchondo B., Yesayan M., Jardon M., Hardie N., Loddenkemper C., Ulasov I., Lesniak M.S. (2013). WNT10B/beta-catenin signalling induces HMGA2 and proliferation in metastatic triple-negative breast cancer. EMBO Mol. Med..

[29] Zha L., Zhang J., Tang W., Zhang N., He M., Guo Y., Wang Z. (2013). HMGA2 elicits EMT by activating the Wnt/beta-catenin pathway in gastric cancer. Dig. Dis. Sci..

[30] Cho J., Ahn S., Yoo K.H., Kim J.H., Choi S.H., Jang K.T., Lee J. (2016). Treatment outcome of PD-1 immune checkpoint inhibitor in Asian metastatic melanoma patients: Correlative analysis with PD-L1 immunohistochemistry. Investig. New Drugs.

[31] Zhao L., Li C., Liu F., Zhao Y., Liu J., Hua Y., Liu J., Huang J., Ge C. (2017). A blockade of PD-L1 produced antitumor and antimetastatic effects in an orthotopic mouse pancreatic cancer model via the PI3K/Akt/mTOR signaling pathway. OncoTargets Ther..

[32] Song M., Chen D., Lu B., Wang C., Zhang J., Huang L., Wang X., Timmons C.L., Hu J., Liu B. (2013). PTEN loss increases PD-L1 protein expression and affects the correlation between PD-L1 expression and clinical parameters in colorectal cancer. PLoS ONE.

[33] Okita R., Maeda A., Shimizu K., Nojima Y., Saisho S., Nakata M. (2017). PD-L1 overexpression is partially regulated by EGFR/HER2 signaling and associated with poor prognosis in patients with non-small-cell lung cancer. Cancer Immunol. Immunother..

[34] Foy J.P., Bertolus C., Michallet M.C., Deneuve S., Incitti R., Bendriss-Vermare N., Albaret M.A., Ortiz-Cuaran S., Thomas E., Colombe A. (2017). The immune microenvironment of HPV-negative oral squamous cell carcinoma from never-smokers and never-drinkers patients suggests higher clinical benefit of IDO1 and PD1/PD-L1 blockade. Ann. Oncol..

[35] Ran X., Yang K. (2017). Inhibitors of the PD-1/PD-L1 axis for the treatment of head and neck cancer: Current status and future perspectives. Drug Des. Dev. Ther..

[36] Chen Y.R., Chen Y.S., Chin Y.T., Li Z.L., Shih Y.J., Yang Y.S.H., ChangOu C.A., Su P.Y., Wang S.H., Wu Y.H. (2019). Thyroid hormone-induced expression of inflammatory cytokines interfere with resveratrol-induced anti-proliferation of oral cancer cells. Food Chem. Toxicol..

[37] Vannini A., Leoni V., Barboni C., Sanapo M., Zaghini A., Malatesta P., Campadelli-Fiume G., Gianni T. (2019). alphavbeta3-integrin regulates PD-L1 expression and is involved in cancer immune evasion. Proc. Natl. Acad. Sci. USA.

[38] Nana A.W., Wu S.Y., Yang Y.S., Chin Y.T., Cheng T.M., Ho Y., Li W.S., Liao Y.M., Chen Y.R., Shih Y.J. (2018). Nano-Diamino-Tetrac (NDAT) Enhances Resveratrol-Induced Antiproliferation by Action on the RRM2 Pathway in Colorectal Cancers. Horm. Cancer.

[39] Konopleva M.Y., Walter R.B., Faderl S.H., Jabbour E.J., Zeng Z., Borthakur G., Huang X., Kadia T.M., Ruvolo P.P., Feliu J.B. (2014). Preclinical and early clinical evaluation of the oral AKT inhibitor, MK-2206, for the treatment of acute myelogenous leukemia. Clin. Cancer Res..

[40] Hu T., Li C. (2010). Convergence between Wnt-beta-catenin and EGFR signaling in cancer. Mol. Cancer.

[41] Paul I., Bhattacharya S., Chatterjee A., Ghosh M.K. (2013). Current Understanding on EGFR and Wnt/beta-Catenin Signaling in Glioma and Their Possible Crosstalk. Genes Cancer.

[42] Gkouveris I., Nikitakis N., Sklavounou A. (2020). p38 Expression and Modulation of STAT3 Signaling in Oral Cancer. Pathol. Oncol. Res..

[43] Leelahavanichkul K., Amornphimoltham P., Molinolo A.A., Basile J.R., Koontongkaew S., Gutkind J.S. (2014). A role for p38 MAPK in head and neck cancer cell growth and tumor-induced angiogenesis and lymphangiogenesis. Mol. Oncol..

[44] Gao Y., Nihira N.T., Bu X., Chu C., Zhang J., Kolodziejczyk A., Fan Y., Chan N.T., Ma L., Liu J. (2020). Acetylation-dependent regulation of PD-L1 nuclear translocation dictates the efficacy of anti-PD-1 immunotherapy. Nat. Cell Biol..

[45] Lin H.Y., Su Y.F., Hsieh M.T., Lin S., Meng R., London D., Lin C., Tang H.Y., Hwang J., Davis F.B. (2013). Nuclear monomeric integrin alphav in cancer cells is a coactivator regulated by thyroid hormone. FASEB J..

[46] Cabodevilla A., Sánchez L., Nintou E., Boiadjieva V., Picatoste F., Gubern A., Claro E. (2013). Cell Survival during Complete Nutrient Deprivation Depends on Lipid Droplet-fueled -Oxidation of Fatty Acids. J. Biol. Chem..

[47] Sharma P., Allison J.P. (2015). Immune checkpoint targeting in cancer therapy: Toward combination strategies with curative potential. Cell.

[48] Spranger S., Bao R., Gajewski T.F. (2015). Melanoma-intrinsic β-catenin signalling prevents anti-tumour immunity. Nature.

