# Supplementary material for: Thyroid Hormone Induces Oral Cancer Growth via the PD-L1-Dependent Signaling Pathway
Source: Cells. 2022 Sep 29;11(19):3050. doi: 10.3390/cells11193050 (PMC9563246; doi:10.3390/cells11193050)
Supplement: Supplementary file 1 [file cells-11-03050-s001.zip › cells-1863033-supplementary.pdf]

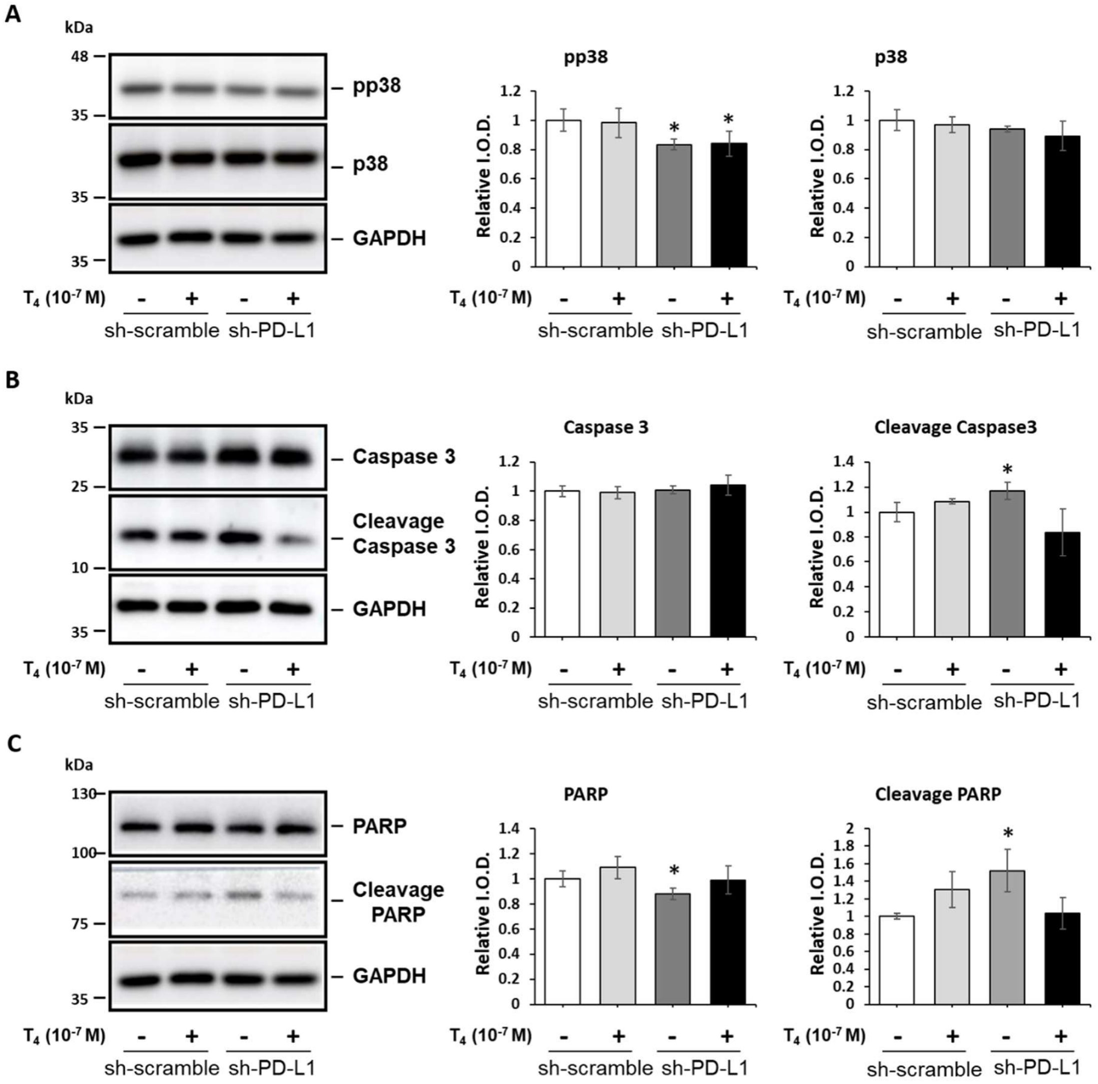


**Figure S1. Deletion of PD-L1 affected proliferation and apoptosis-related protein accumulation in oral cancer cells.** Activation of (A) p38, (B) Caspase 3, and (C) PARP was altered by PD-L1 knockdown. Oral cancer OEC-M1 cells were seeded in six-well trays and were transfected with either PD-L1 shRNA or a scrambled plasmid (0.5 μg/well). Prior to treatment, cells were refed with hormone-stripped FBS-containing medium and treated with $T_4$ ($10^{-7}$ M) for 24 h. Cells were harvested, and Western blotting analyses of pp38, p38, Caspase 3, and PARP were conducted. Data are presented as the mean ± SD. Results are from three independent studies (n = 3) * $p < 0.05$, compared to the control. I.O.D., intensity of the optical density.

**Supplementary Materials and Methods**

Oral cancer OEC-M1 cells were seeded (105 cells/well) in six-well tissue culture plates, grown overnight, and maintained in the absence of antibiotics for 24 h before transfection. Just prior to transfection, the culture medium was removed, and cells were washed once with phosphate-buffered saline (PBS) and then transfected with PD-L1 shRNA (CD274, clone ID: TRCN0000056916) or a scrambled plasmid (TRC1.Scramble, clone ID: ASN0000000004) (0.5 μg/well, RNAi Core Facility, Academia Sinica, Taipei, Taiwan) by Lipofectamine 3000 Kit (ThermoFisher Scientific, L3000001) in Opti-MEM I medium according to the manufacturer's instructions. After transfection, cultures were incubated at 37 °C for 6 h and then placed in a fresh culture medium for additional 48 h. Transfected cells were then treated with 10-7 M T4 24 h for Western blotting analyses

For the Western blotting analyses, cells were lysed, and extracted protein samples were separated by 10% - 15 % sodium dodecylsulfate polyacrylamide gel electrophoresis (SDS-PAGE). A 15-μg quantity of protein was loaded into each well, and samples were separated by electrophoresis at 100 V for 2 h. Separated proteins were transferred from the polyacrylamide gel to Millipore Immobilon-PSQ Transfer polyvinylidene difluoride (PVDF) membranes (Millipore, Billerica, MA, USA) using Mini Trans-Blot® Cell (Bio-Rad Laboratories, Hercules, CA, USA). Membranes then were incubated in NaCl/Tris blocking buffer containing 2% bovine serum albumin (BSA). Membranes were incubated with primary antibodies for pp38 (Cell Signaling, #4511), p38(Cell Signaling, #8690), Caspase 3 (GeneTex, GTX110543), and PARP (GeneTex, GTX100573), or GAPDH (proteintech, 60004-1-Ig) overnight at 4 °C. After 0.1% TBST washed, Proteins were detected with horseradish peroxidase (HRP)-conjugated secondary antibodies (Jackson ImmunoResearch, #115-035-003 and #111-035-003) and the Immobilon™ Western HRP Substrate Luminol Reagent (Millipore, WBKLS0500). Western blots were visualized and recorded with an Amersham Imager 600 (GE Healthcare Life Sciences, Pittsburgh, PA, USA). The densitometric analysis of Western blots was conducted using ImageJ 1.47 software (National Institutes of Health, Bethesda, MD, USA) according to the software instructions.
